# The Zinc Dyshomeostasis Hypothesis of Alzheimer's Disease

**DOI:** 10.1371/journal.pone.0033552

**Published:** 2012-03-23

**Authors:** Travis J. A. Craddock, Jack A. Tuszynski, Deepak Chopra, Noel Casey, Lee E. Goldstein, Stuart R. Hameroff, Rudolph E. Tanzi

**Affiliations:** 1 Department of Physics, University of Alberta, Edmonton, Alberta, Canada; 2 Division of Experimental Oncology, Cross Cancer Institute, University of Alberta, Edmonton, Alberta, Canada; 3 The Chopra Center for Well-Being, Carlsbad, California, United States of America; 4 Center for Biometals & Metallomics, Alzheimer's Disease Center, Boston University School of Medicine, Boston, Massachusetts, United States of America; 5 Departments of Anesthesiology and Psychology, Center for Consciousness Studies The University of Arizona Health Sciences Center, Tucson, Arizona, United States of America; 6 Genetics and Aging Research Unit, Department of Neurology, Massachusetts General Hospital- East, Charlestown, Massachusetts, United States of America; New York State Institute for Basic Research, United States of America

## Abstract

Alzheimer's disease (AD) is the most common form of dementia in the elderly. Hallmark AD neuropathology includes extracellular amyloid plaques composed largely of the amyloid-β protein (Aβ), intracellular neurofibrillary tangles (NFTs) composed of hyper-phosphorylated microtubule-associated protein tau (MAP-tau), and microtubule destabilization. Early-onset autosomal dominant AD genes are associated with excessive Aβ accumulation, however cognitive impairment best correlates with NFTs and disrupted microtubules. The mechanisms linking Aβ and NFT pathologies in AD are unknown. Here, we propose that sequestration of zinc by Aβ-amyloid deposits (Aβ oligomers and plaques) not only drives Aβ aggregation, but also disrupts zinc homeostasis in zinc-enriched brain regions important for memory and vulnerable to AD pathology, resulting in intra-neuronal zinc levels, which are either too low, or excessively high. To evaluate this hypothesis, we 1) used molecular modeling of zinc binding to the microtubule component protein tubulin, identifying specific, high-affinity zinc binding sites that influence side-to-side tubulin interaction, the sensitive link in microtubule polymerization and stability. We also 2) performed kinetic modeling showing zinc distribution in extra-neuronal Aβ deposits can reduce intra-neuronal zinc binding to microtubules, destabilizing microtubules. Finally, we 3) used metallomic imaging mass spectrometry (MIMS) to show anatomically-localized and age-dependent zinc dyshomeostasis in specific brain regions of Tg2576 transgenic, mice, a model for AD. We found excess zinc in brain regions associated with memory processing and NFT pathology. Overall, we present a theoretical framework and support for a new theory of AD linking extra-neuronal Aβ amyloid to intra-neuronal NFTs and cognitive dysfunction. The connection, we propose, is based on β-amyloid-induced alterations in zinc ion concentration inside neurons affecting stability of polymerized microtubules, their binding to MAP-tau, and molecular dynamics involved in cognition. Further, our theory supports novel AD therapeutic strategies targeting intra-neuronal zinc homeostasis and microtubule dynamics to prevent neurodegeneration and cognitive decline.

## Introduction

Alzheimer's disease (AD) is the major cause of dementia and a leading cause of death in the elderly. Early symptoms include inability to form new memories, confusion, and mood swings. Clinical progression inevitably involves cognitive dysfunction, neuropsychiatric disturbances, psychosocial derailment, and death. Although numerous therapeutic approaches have been implemented, no clinically useful disease-modifying treatments are currently available. With tens of millions of AD patients worldwide requiring care and accelerating AD epidemiological trends, the disease presents medical, social and economic problems of global proportion.

The brains of patients affected by AD have two types of neuropathological lesions. In AD, extracellular deposition of the ∼4 kDa amyloid-β (Aβ) peptide derived from the amyloid precursor protein (APP), leads to amyloid plaques and neurotoxic oligomers that impair long term potentiation (LTP) and synaptic function [1]. At the intracellular level, cortical neurons in the AD brain accumulate hyper-phosphorylated tau, a microtubule-associated protein (MAP), which triggers formation of neurofibrillary tangles (NFTs) [1]. Neurons in AD brain also demonstrate impaired axonal transport, motor protein transport along axonal microtubules (MTs), and compromised MT networks [2].

While all four established AD genes lead to excessive accumulation of Aβ peptide in the brain, resulting β-amyloid deposition is necessary but not sufficient for the onset of AD. Dementia and neurodegeneration initiated by β-amyloid deposition require tauopathy and microtubule destabilization, including NFT formation [3]. How β-amyloid accumulation in AD brain leads to NFT pathology remains unknown. Zinc has previously been shown to promote the aggregation of β-amyloid, which sequesters the metal and promotes local zinc dyshomeostasis in the vicinity of β-amyloid deposits [4]. Here we argue that Aβ-mediated zinc sequestration outside neurons depletes intra-neuronal zinc stores leading to MT destabilization, NFT formation, and neuronal degeneration, neuronal degeneration. Aβ aggregation may also result in excessive intra-neuronal levels of zinc. In this model of AD, MT destabilization is the primary cause of tau release and hyperphosphorylation, NFT formation, neuronal dysfunction, and dementia.

In this article we first review the role and relevance of neuronal microtubules to memory and cognitive functions affected by AD. We then report on three approaches we employed to test our hypothesis of a zinc connection between β-amyloid, microtubules and AD pathology.

1. Using molecular modeling of tubulin, the component protein of MTs, we identified specific, high-affinity electrostatic zinc binding sites that influence side-to-side tubulin interaction, the sensitive link in microtubule polymerization and lattice stability. This suggests that insufficient levels of intraneuronal zinc would destabilize MTs, free tau proteins, and disrupt intra-neuronal cytoskeletal architecture, thereby impairing memory and cognition. Additionally, we show how excessive intra-neuronal zinc can disrupt MT polymerization through aberrant tubulin-tubulin binding.
2. We performed kinetic modeling showing zinc distribution in extra-neuronal Aβ deposits can cause intra-neuronal zinc depletion, reduced zinc binding to microtubules, and microtubule disruption.
3. We used metallomic imaging mass spectrometry (MIMS) to show anatomically-localized and age-dependent zinc dyshomeostasis in specific brain regions of Tg2576 transgenic, AD-model mice, brain regions (e.g. hippocampus, dentate gyrus, subiculum, and cortical layer II) associated with memory, cognition and NFT pathology.

We present a comprehensive theory of AD pathogenesis in which β-amyloid plaque formation promotes intra-neuronal zinc depletion, and/or excess intra-neuronal zinc, to levels, which disrupt MTs, promote NFTs, and promote cognitive impairment. This in turn suggests novel AD therapeutic strategies aimed at restoring intraneuronal zinc homeostasis and stabilizing microtubule lattice structure.

### Microtubules and Memory

Cytoskeletal polymers including actin, neurofilaments and MTs structurally and dynamically organize the interiors of neurons, and other cells. The most rigid cytoskeletal component, MTs self-assemble from tubulin proteins to form microns-long hollow cylinders with outer diameters of 25–26 nm and inner diameters of 15 nm. MTs typically consist of 13 linear chains of tubulin dimers called protofilaments, which align side-to-side resulting in hexagonal lattices of tubulin dimers comprising the MT cylindrical wall. MTs have an electric and functional polarity. MT assembly and subsequent binding of various MAPs determine cell form and function, including formation and maintenance of neuronal axons, dendrites and synapses.

**Figure 1:**
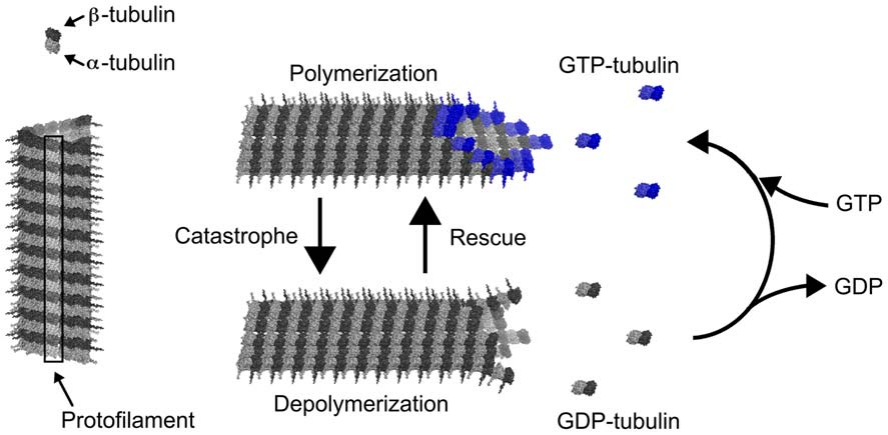
Microtubule assembly/disassembly. Each longitudinal protofilament of an MT is made up of α and β tubulin subunit heterodimers. Assembly of MTs depends on addition of ‘GTP-tubulin’. Top, right: blue dimers with both exchangeable and non-exchangeable sites occupied by GTP will continue to add to assembling MTs. Bottom, right: dimers at MT edge which hydrolyze GTP to GDP flex and separate protofilaments from MT, curved protofilaments resembling ‘rams horns’.

Tubulin is a heterodimer consisting of 55 kD α-tubulin and β-tubulin monomers (see Fig. 1), highly conserved in eukaryotic cells. Tubulin polymerization depends on various physical (temperature) and chemical (pH, concentration of ions) factors. Under normal conditions, tubulin assembly depends on GTP occupancy. Each tubulin dimer can bind two guanosine-tri-phosphate (GTP) one at a non-exchangeable site on the alpha monomer, another at an exchangeable site on the beta monomer at which GTP can hydrolyze to guanosine-di-phosphate (GDP), imparting energy and conformational flexing to the underlying tubulin. In assembling MTs, exchangeable GTP tubulin on the exposed end will undergo hydrolysis within a brief time if not covered by another tubulin. Thus MTs, which continue to grow and add GTP tubulin, are stable. However if assembly stalls, GTP hydrolysis at the MT exposed end occurs, and triggers rapid disassembly (termed MT ‘catastrophes’) (Figure 1).

Usually, assembly proceeds by formation of protofilaments, which then align side-to-side, and disassembly occurs via separation of side-to-side protofilaments. Because GTP hydrolysis to GDP causes conformational flexing of the tubulin dimer, protofilaments separate and curve away from one another in a pattern referred to as “ram's horns” (Figure 1). In many cells and conditions, MTs exist in cycles of assembly/disassembly called ‘dynamic instability’, useful in probing and retreating in cell growth and development. Some MTs assemble at one end and disassemble at the other, a process called ‘treadmilling’. However in brain neurons, specialized MAPs called ‘STOP’ proteins cap MT ends, preventing GTP hydrolysis, dynamic instability and treadmilling. MTs in brain neurons are stable, and potential sites for memory and cognitive processes in normal healthy conditions.

**Figure 2:**
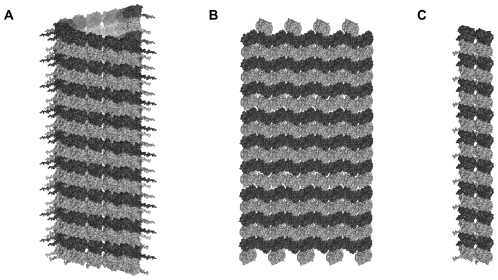
Tubulin assembly depends on ambient conditions. (A) Cylindrical ‘B-lattice’ MT composed of 13 protofilaments, formed under physiological conditions. (B) In excess zinc ion concentration, tubulin forms flat sheets rather than cylinders. (C) Tubulin can form double protofilaments and other types of assemblies described in [7].

However abnormal physiological or biochemical conditions can affect MT assembly, e.g. resulting in aberrant formation of closely or widely spaced MTs, tubulin sheets, rings/ribbons or various other structures [5]–[7], (Figure 2). One important factor among these is zinc ion concentration. Whereas low/moderate levels of zinc enhance tubulin polymerization, excessive zinc levels induce tubulin to form flat sheets rather than cylinders (Figure 2B).

In all eukaryotic cells, MTs and cytoskeletal components establish cell shape and enact movement, including mitosis, growth and motility, by their own assembly and coordinated activities of MAPs and actin filaments. In highly asymmetrical neurons, microtubules play an especially important role in cell morphology; establishing and maintaining elongated axons, dendrites and their synapses. In neuronal axons, MTs are continuous and of the same polarity, while in dendrites MTs are interrupted and of mixed polarity. In both axons and dendrites, various MAPs interconnect MTs into networks, scaffolding of the neuronal and synaptic architecture. Once formed, synapses are regulated by transport of synaptic components along MTs by motor proteins kinesin and dynein. Material synthesized in the cell body may require transport through a highly branched dendritic tree, switching from MT to MT numerous times. The motor protein mechanical transport requires chemical energy (ATP hydrolysis), but the guidance mechanism, which brings specific precursors to regulate specific synapses, is unknown. Recently tau, the MAP involved in AD, was shown to act as a traffic signal, discharging motor proteins and their cargo at specific locations on the MT lattice network [8]. Thus, specific binding sites and patterns of tau (and other MAPs) on MT lattices can encode information involved in synaptic plasticity, i.e. memory.

Memory is generally considered to depend on synaptic plasticity, sensitivity of specific synapses in neuronal networks, guiding network activity and computation. However, synaptic proteins and other components last only hours to days, being recycled by materials transported by motor proteins along microtubules. Another site for memory encoding and storage is required. Several lines of evidence point to microtubules.

Cronly-Dillon and co-workers [9] showed that when baby rats first open their eyes, genes in visual cortex suddenly begin producing vast quantities of tubulin. When the rats are 35 days old, the critical phase for learning is over and tubulin production is drastically reduced. They concluded tubulin turnover and microtubule activity are involved in synaptic plasticity, learning and memory.

Dendrite-specific MAP2 is particularly important in memory consolidation, with reduced levels or activity in situations of impaired memory. For example memory deficits correlate with decreased levels of MAP2 in hippocampus [10]–[12], and cerebral hypoperfusion with impaired cognitive performance results in decreased MAP2 [13]. The senescence-accelerated (SAMP10) mouse strain, which exhibits learning and memory deficits, expressed less cortical MAP2 [14]. Transgenic mice with altered MAP2 showed impairments of contextual memory and reduced capacity for phosphorylation of MAP2 [15].

Increased learning correlates with enhanced MAP2 activity. Fear conditioning produced clear changes in MAP2 immunohistochemical staining in regions of the cerebral cortex or hippocampus [16]–[18]. Enhanced MAP2 turnover [19] was found in pyramidal cells of the hippocampus with contextual learning. Similar learning-related changes in MAP2 followed avoidance training [20]. MAP2-mediated reorganization of MT networks correlates with memory.

Memory and learning are studied through in vivo models of ‘long-term potentiation’ (LTP), e.g. in hippocampal slice. Induced high frequency pre-synaptic stimulation results in prolonged, enhanced post-synaptic sensitivity, presumably correlating with Hebbian learning in synapses. During LTP, MAP1B phosphorylation [21] and local concentrations of mRNA for MAP2 and for Ca^2+^-calmodulin-dependent kinase II (CaMKII) increase [22]. CaMKII is responsible for phosphorylating MAP2, enhancing synaptic response [23].

A key step in LTP occurs when calcium ions enter the post-synaptic neuron and activate the hexagonal holoenzyme CaMKII, causing it to transform and extend 6 kinase domains above and below the main body. These kinase domains can phosphorylate intra-neuronal targets for memory encoding and storage. Hameroff et al [24], [25] showed that the geometry of 6 extended CaMKII kinase precisely matches microtubule lattices, enabling CaMKII to phosphorylate 6 bits of information (one byte) to a small region of a MT lattice. Previous theoretical work had suggested various forms of information processing in microtubules [26]–[28]. MT-depolymerizing agents cause amnesia, and LTP with protein synthesis involves formation of new MT tracks between stimulated synapses and the nucleus in the soma. MTs are likely candidates for memory storage. Tubulin is a candidate to manifest interactive bit-like information states.

Generally considered as merely bone-like structural support, MTs have been proposed to also function as intra-cellular information processing devices in which tubulin states represent and process fundamental information bits (by phosphorylation, conformation, dipoles) within MT lattices acting as computational automata [29]–[31]. Individual tubulin states within MT lattices may be further modified/programmed not only by phosphorylation, but also by post-translational modifications and binding of various ligands and MT-associated proteins. Such information could also be transferred to particularly long-lasting and stable parallel cytoskeletal structures including neurofilaments, and read out, or implemented in several ways. Post-synaptic dendritic/somatic MTs participate in integration of inputs, helping to determine axonal firing as outputs, and also regulating synaptic plasticity. Specific patterns of phosphorylation would likely modify and influence dynamical MT functions governing neurite extension, motor protein transport and binding sites for MT-associated proteins determining synaptic architecture and plasticity. MTs are logical sites for intra-neuronal memory processing.

### MAPs, Tau, Neurofibrillary Tangles and Alzheimer's Disease

Intraneuronal pathological lesions in AD involve hyper-phosphorylated tau which first oligomerizes, then forms insoluble paired-helical filaments (PHFs) and finally NFTs, which also may include MAP-2. Concomitant with PHF and NFT formation, MTs also depolymerize. The sequence of these events remains uncertain.

MAP-tau is one of many microtubule-associated proteins in brain neurons (Table 1). At the neuronal cellular level, MTs in neuronal cell body/soma and dendrites bind MAP2 preferentially, whereas MTs in axons prefer MAP-tau. Nevertheless, hyper-phosphorylated MAP-tau, PHFs and NFTs are highly concentrated in the somato-dendritic part of the neuron, with relative sparing of axons.

**Table 1:** Microtubule-binding proteins and their functions.

Protein	Functions	References
*Stabilizing MAPs*		
MAP1A	Neural development; stabilization of MTs in axons and dendrites	[105], [106]
MAP1B	Neural development; stabilization of MTs in axons and dendrites	[105]–[108]
MAP2A	Neural development; stabilization of MTs in dendrites; signal transduction	[105], [109]–[110]
MAP2B	Neural development; stabilization of MTs in dendrites, signal transduction	[105 109–110]
MAP2C	Early neural development	[105], [109]–[111]
MAP-tau	Neural development; stabilization of MTs in axons; axonal transport	[105], [109], [111]
*Proteins related to specific neurodevelopmental disorders*		
ASPM*	Responsible for brain enlargement	[112]
DCX**	Neural development; cortical neuron migration	[112]
LIS1†	Neural development; cortical neuron migration	[112]
CLIP-115††	Regulates MT dynamics by binding to tips of growing MTs	[113]
*Motor proteins*		
Dynein	Retrograde transport in the axon; transport to minus ends of MTs in dendrites	[114], [115]
Kinesin	Anterograde transport in the axon; transport to plus ends of MTs in dendrites	[114], [115]

* ASPM: abnormal spindle-like protein, microencephaly-associated.

** DCX: doublecortin.

† LIS1: lissencephaly-1.

†† CLIP-115: cytoplasmic linker protein-115.

Woolf et al. suggested an imbalance of MAPs throughout brain neurons lead to cytoskeletal breakdown in AD [32]. MAP-tau over-expression in hippocampal neurons results in increased MAP-tau levels in dendrites and loss of dendritic spines [33], suggesting that MAP-tau competes with other MAPs affecting MT dynamics. In the AD brain, expression of MAP2 (found primarily in dendrites and implicated in learning and memory) is decreased while MAP-tau levels remain normal [34], and MAP2 immuno-staining is negatively correlated with NFT levels [35]. These results suggest an inverse relationship between MAP2 levels and the presence of NFTs. NFTs and associated MT depolymerization in soma and dendrites are most debilitating to neuronal function, consistent with other approaches suggesting cognition, memory and consciousness occur primarily in soma and dendrites rather than in axonal firings [36].

**Figure 3:**
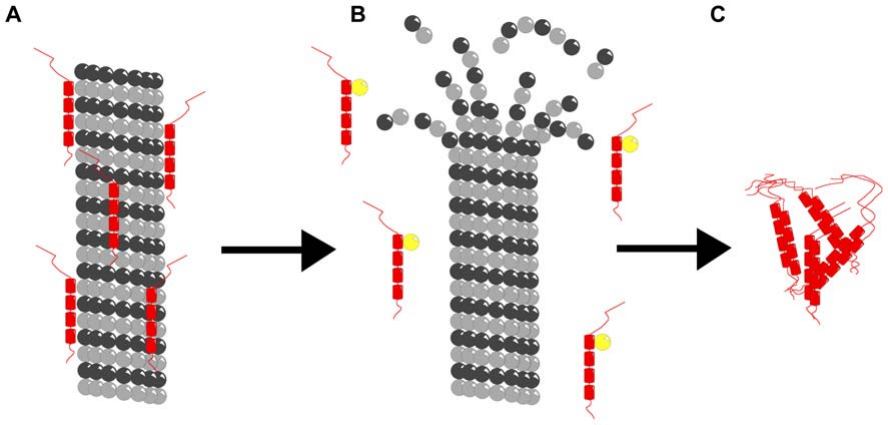
Schematic illustration of MT depolymerization in AD neurons. (A) Microtubule stabilized by MAP-tau (red). (B) Destabilized microtubule and free MAP-tau with additional phosphorous (yellow). (C) Neurofibrillary tangles formed of hyper-phosphorylated MAP-tau.

Prior to displacement from MTs, MAP-tau is linear, approximately 16 nanometers in length, contacting and extending over two or more dimers along a protofilament (Figure 3). Functionally, MAP-tau has been thought to stabilize MTs, but, as mentioned above, recent evidence suggests that tau acts like a motor-protein traffic signal, causing the release of synaptic cargo and regulating synaptic plasticity [8] This implies tau-binding locations are encoded as information in MT lattices.

Current dogma suggests MAP-tau is hyper-phosphorylated on MTs, leading to MT destabilization and aggregation of MAP-tau (and other MAPs) into insoluble protein clusters. The tau protein then coils into paired helical filaments (PHFs), which adopt a β-conformation, eventually transforming the aggregate into NFTs (Figure 3). But evidence is inconsistent, and molecular events triggering NFT formation remain unclear.

Thus the following are open questions. Does hyperphosphorylation of MAP-tau on MTs lead to MT disruption, removal of MAP-tau from MTs, and NFTs? Or does MT depolymerization initiate the process, causing release of MAP-tau, which is then hyper-phosphorylated to form NFTs? Which of these cause cognitive defects? How can they be stopped?

Hyper-phosphorylated MAP-tau and NFTs separated from MTs can dramatically alter MT dynamics, for example blocking MT assembly. In experiments where hyper-phosphorylated MAP-tau isolated from Alzheimer's disease brain is added to mouse embryonic fibroblasts, MT polymerizations is impaired, suggesting that MAP-tau hyper-phosphorylation is responsible for the MT dysfunction in AD.

However NFTs and hyper-phosphorylated tau may exacerbate MT defects already present in AD patients' brains. Some studies indicate MT dysfunction is a primary problem in AD, and not merely a result of NFTs compromising neuronal integrity [2]. When neurons in AD brain were evaluated for MT abnormalities, even seemingly healthy neurons (i.e. without PHFs or NFTs) in AD brains exhibited MT defects, with MT numbers and total MT lengths decreased compared to those in neurons from controls. Neurons in AD brains also demonstrate impaired motor protein transport along MTs, and compromised MT-MAP networks [2]. Recent evidence suggests that preventing neurofibrillary tangles is more likely to protect against dementia and cognitive impairment than is targeting β-amyloid plaques [3].

Here, we assess whether the primary event driving NFT formation is MT instability due to reduced zinc concentration in neuronal cytoplasm. We examine in detail the theoretical binding sites for zinc on tubulin, the constituent protein of microtubules.

### Tubulin, Zinc, and Microtubule Polymerization

MTs are polymers of tubulin. The atomic structure of tubulin was resolved to within 3.7 Å resolution from electron crystallography by Nogales et al [37], and later refined [38] to 3.5 Å. Tubulin has a β- sheet core, surrounded by α-helices and a Rossmann-fold nucleotide-binding domain at the N-terminal region. It also has an intermediate domain containing a mixed, four-strand β sheet and three helices. A third domain consists of two anti-parallel helices that cross the first two domains.

**Figure 4:**
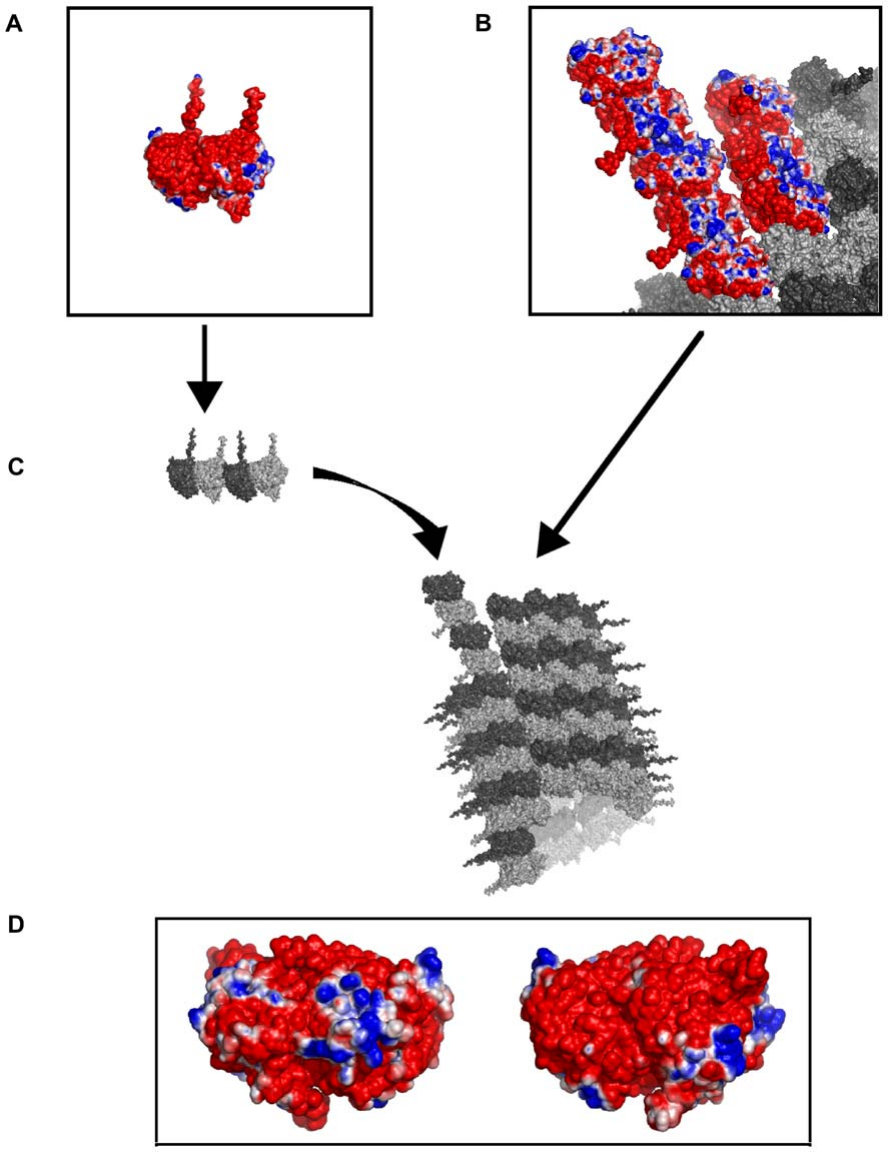
Charge distribution on the surface of an MT following [72]. Red – negative charge at a potential of ^−^1 kT/e, Blue – positive charge at a potential of 1 kT/e. (A) Close up, a single tubulin dimer electrostatic map. (B) Close up, Electrostatic attraction between tubulin dimers during MT polymerization. (C) Microtubule polymerization. (D) Opposing faces of adjacent dimers showing complimentary electrostatic patterns.

As shown in Figure 4, the tubulin C-terminus, known as the C-terminal ‘tail’, extends outward from the MT surface on each monomer with significant negative electric charge (as much as 40% of the total monomer charge). These negative charges on each C-terminus are responsible for its extended conformational state since the surface charge is also negative leading to Coulomb repulsion. Electric charge distribution is crucial to the organization of tubulin dimers into a MT lattice as can be seen in Figure 4 for the α-β dimers.

Zinc is an abundant transition metal found in large concentrations in mammalian brain with some synapses in the hippocampus containing concentrations as high as 150 µM. Of this, 85–90% is bound to zinc metalloproteins, the remaining 10–15% either loosely bound or free in presynaptic vesicles [39]–[41]. Cytosolic free concentration of zinc in cultured neurons is generally subnanomolar, however in pathological conditions levels of free zinc ion (Zn^2+^) can change via several pathways [42]. Free zinc is a potent killer of neurons and glia with extended exposure to as little as 100 nM leading to neuronal death [42]. However critical amounts of bound zinc are essential for various enzymes and biochemical activities.

Zinc binds to tubulin and affects its polymerization into MTs [43], [44]. For low zinc to tubulin ratios, polymerization of tubulin into MTs is enhanced both *in vitro* and *in vivo*. However at zinc to tubulin ratios exceeding 3∶1, tubulin polymerization becomes aberrant, forming flat sheets rather than cylinders [45]–[48]. This suggests the tubulin dimer optimally binds 1 to 3 zinc ions.

Axons incubated with low zinc concentrations (5 µM) show increased motor protein transport along microtubules; cultured neurons incubated in high zinc concentrations (0.1–1.0 mM) have structural damage and decreased motor protein activity along MTs [49]–[51]. Dietary zinc deficiency results in the impairment of tubulin polymerization into MTs [52]–[54]. It appears MTs function optimally at an intermediate range, a window of zinc concentration.

Zinc deficiency may also lower levels of α- and β-tubulin, MAP2 expression and impair MT polymerization [55], [56]. Zinc deficiency effects on lowered MT polymerization rates are apparently mediated through decreased electrostatic attraction between tubulin dimers, as well as decreased expression of MAP-tau, MAP2, and potential lowering of MAP-tau binding to MTs. We focus on zinc binding to tubulin.

In equilibrium conditions, Eagle et al. [57] found 65 zinc binding sites on tubulin for zinc, 10 of which were so-called high affinity sites with K_d_ ∼2.6 µM, and 55 were low affinity sites with K_d_ ∼55 µM. Under assembly conditions 3 sites of K_d_ ∼0.9 µM and 17 sites K_d_ ∼16 µM were found. However, Hesketh found zinc-binding sites with 110 µM [58]. The location of these sites remains unknown. The only identified zinc-tubulin binding site is the putative sheet-inducing site from the tubulin crystal structure [37], [38]. GTP concentration affects zinc binding [57], and the exchangeable GTP site on tubulin is altered by zinc [59]. Bound zinc ions also alter the colchicine-binding site on tubulin [60], and zinc may alter tubulin phosphorylation [61].

## Methods

### Molecular Modeling of Theoretical Zinc Binding Sites on Tubulin

#### Tubulin and MT Modeling

The Protein Data Bank (PDB) [62] crystal structure of bovine brain tubulin 1JFF [38] was repaired via homology modeling by adding missing residues from 1TUB [37] using MODELLER 9V6 [63]. The repaired 1JFF dimer was solvated, neutralized and energy-minimized using the molecular dynamics simulator NAMD [64], developed by the Theoretical and Computational Biophysics Group in the Beckman Institute for Advanced Science and Technology at the University of Illinois at Urbana-Champaign. Using this dimer, MT A and B lattice structures were built with PYMOL 0.99rc6 [65] using MT geometry described in Li et al. [66] and Sept et al. [67].

#### Tubulin-Zinc Binding Site Prediction

To characterize zinc-binding sites on tubulin the minimized repaired 1JFF structure was run through the robust zinc protein-binding site prediction algorithm FEATURE: Metals [68] FEATURE: Metals structurally predicts zinc-binding sites based on coordination geometry trained on positive (known zinc binding sites) and negative (non-metal binding regions with characteristics of zinc binding sites) samples in conjunction with additional biophysical and biochemical properties averaged around the site of interest, with a 73.6% in unbound known proteins structures and 65.5% in protein structures determined from homology models. Low stringency results were spatially clustered via a density-based spatial clustering of applications with noise (DBSCAN) [69] with a minimum group size of 1 and a nearest-neighbor distance of 1.5 Å.

#### Electrostatic Analysis

To analyze the electrostatics of the systems, hydrogens were added, and protonation states set at pH 7 with PROPKA [70], via PDB2PQR [71], [72] for both the tubulin dimer and MT lattice structures. The Poisson-Boltzmann equation was solved for the structures in given arrangement with the Adaptive Poisson-Boltzmann Solver (APBS) [73] with less than 1 Å spacing. To investigate the effect of the zinc ions of the electrostatic profile of tubulin the Poisson-Boltzmann equation was solved for the tubulin dimer both with zinc ions, at the top six sites. Zinc ions were given a charge of +2e and radius of 1.10 Å. Charges on the key binding residues were modified to allow zinc binding, partially canceling the zinc charge following the cationic dummy atom approach (CaDA) [74]–[76]: Cysteine and histidine residues were used in their anionic form, and glutamic acid and aspartic acid were used in their neutral form.

All illustrations were created using PYMOL 0.99rc6 [65].

### Metallomic Imaging Mass Spectrometry (MIMS Mapping) of Elemental Zinc Distribution in the Brain of Aged Tg2576 AD Transgenic Mice Compared to Wild Type Controls

Analysis of murine brain specimens by MIMS was conducted at the Center for Biometals and Metallomics, Boston University School of Medicine, Boston, MA, USA. A double-focusing magnetic sector field ICP-MS (Element XR, Thermo Scientific, Waltham, MA, USA) was custom hyphenated to a Nd-YAG laser with frequency quadrupling to attain 213 nm output (LSX 213, Cetac Technologies, Omaha, NE, USA). Laser pulse duration was 5 ns with a maximum pulse frequency of 20 Hz. Energy density was constant at a maximum of 4 mJ. Spot size was varied between 5 µm (high resolution) and 50 µm (low resolution). Laser-generated aerosol was transported from a custom-designed laminar flow cryogenically-cooled laser ablation cell (Geomed Analytical, University of Massachusetts, MA, USA) maintained at −15 to −25°C ±0.1°C to the sector field ICP mass spectrometer by a carrier gas mixture of helium and argon at constant flow rates of 1 L min^−1^ and 0.8 L min^−1^, respectively. The ICP-mass spectrometer was synchronized with the laser ablation platform using an external contact closure trigger. Ions generated in the ICP were extracted into the mass spectrometer and separated according to mass-to-charge ratio. The ICP torch was shielded with a grounded platinum guard electrode. ICP-mass spectrometer analytical optimization and calibration was performed using ^59^Co, ^139^La, and ^232^Th line-scanning ablation of a reference glass standard (SRM 612, National Institute of Standards and Technology, Gaithersburg, MD, USA). Analytical conditions were optimized to ensure that oxide formation was <1% based on ratio measurement of ^232^Th^16^O and ^232^Th. The hyphenated laser ablation-assisted MIMS system generated a signal of <7% RSD for a 2 min continuous line scan at a spot size of 50 µm, line scan velocity of 50 µm/s, and frequency of 20 Hz. The plasma conditions for MIMS analysis of brain specimens were optimized for ^63^Cu, ^66^Zn and ^70^Zn. Brain specimen positioning was accomplished by coordination with a multi-line map grid with 50 µm spacing between adjacent lines. After MIMS analysis, data sets were exported to customized Matlab program (Matlab 2010, Mathworks, MA, USA) for raw data processing, analytical quantitation, and two-dimensional mapping. Color intensity image maps were produced for each isotope by converting the temporally acquired sector field ICP-mass spectrometry signal (counts/second) into distance (microns) travelled by the laser ablation platform.

We deployed high resolution MIMS mapping to test the hypothesis that increased AD-linked Aβ accumulation in aged Tg2576 AD transgenic mice is associated with local zinc dyshomeostasis in zinc-enriched brain regions affected by AD neuropathology. To conduct this study, we used aged Tg2576 AD transgenic mice [77] that carry a transgene construct containing the human Swedish mutant APP (APPswe).

This study was approved following full board review by the Boston University School of Medicine Institutional Animal Care & Use Committee (IACUC) and approved as protocol AN-15088.2011.03 effective 6/09/2011 (expiration 3/18/2012).

## Results

### Theoretical zinc binding sites on tubulin

**Figure 5:**
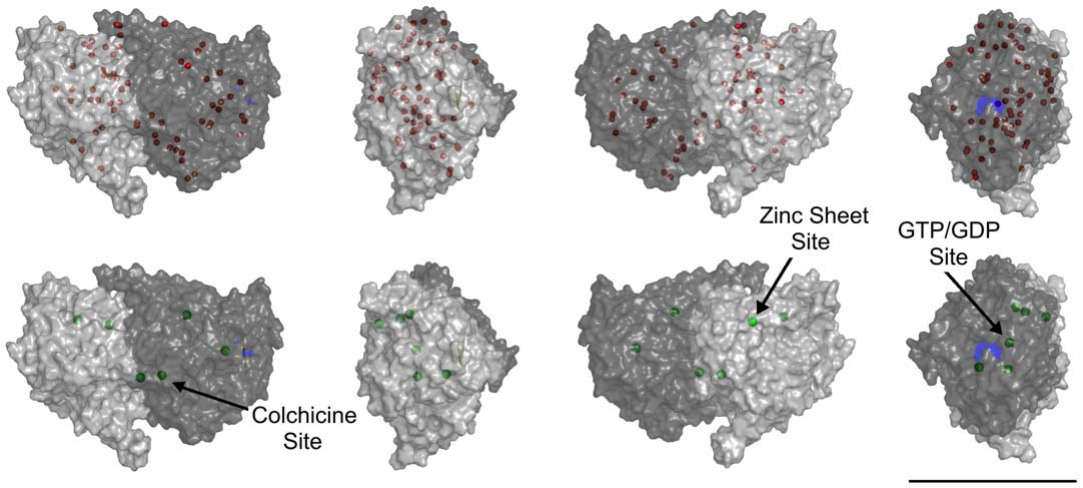
Unique zinc binding sites on tubulin. Top row - all 64 predicted sites at low stringency (red). Bottom row - top six predicted sites at high stringency (green). Light grey - α-tubulin, Dark grey - β-tubulin, Blue - Exchangeable GTP/GDP site. Tubulin is rotated in 90° increments moving from left to right. Scale bar corresponds to 5 nm.

Under low stringency the zinc prediction program yields 591 non-unique hits. Clustering these by spatial density [69] reveals 64 unique zinc sites, however 6 of them are on the C-terminal tails and, due to their flexibility and high sequence variability between isoforms, cannot be taken as definitive. This leaves 58 predicted sites in agreement with the results of Eagle et al. [57]. Of the 58 predicted, one is at the exchangeable GTP site, 4 at the non-exchangeable GTP site, one at the colchicine site, 2 at the taxol site, and 3 are within 3 Å of SER and THR residues which may be phosphorylated in the C-terminal region [78]. Additionally, two of the residues in the putative zinc site given by the zinc-induced sheet crystal structure [38] are also predicted. Under the highest stringency, only 6 sites are predicted, and these include the colchicine-binding site, the exchangeable GTP/GDP site, and one residue given in the putative zinc-induced sheet site (see Figure 5).

Cysteine, histidine, aspartic acid and glutamic acid amino acid residues account for ∼97% of all zinc-binding amino acids [79]. Typically, four of these residues are capable of neutralizing the charge of the zinc ion, binding it in place. However, the amino acid residues surrounding the predicted zinc sites on a single tubulin dimer do not contain enough of these key residues to stabilize the zinc ion. Hydroxide ions in water are capable of reacting with zinc, potentially stabilizing zinc at the predicted sites. Another possibility is that unbalanced charge of zinc at these sites plays a role in electrostatic protein-protein interactions, namely MT polymerization.

**Figure 6:**
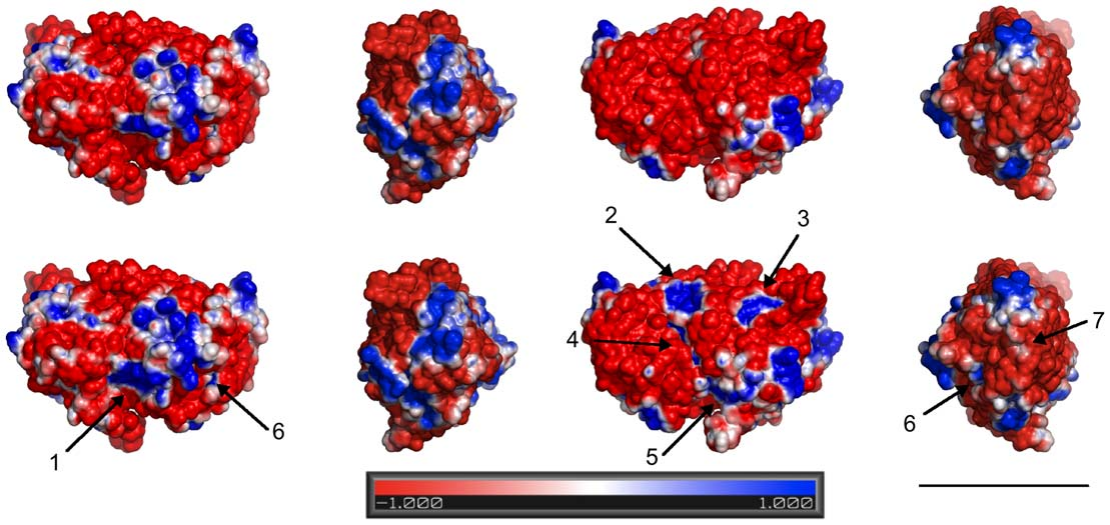
Effect of the top six zinc binding sites on the tubulin electrostatic. Rotation of the tubulin dimer in 90° increments from left to right. Top row – tubulin without zinc. Bottom row – tubulin with zinc at the top six sites, arrows indicate regions of change. Blue – positive charge, Red – negative charge, White – electrostatically neutral. Potential map ranges from −1 kT/e to 1 kT/e. Scale bar corresponds to 5 nm.

The electrostatic profile of the tubulin dimer is critical to MT polymerization dynamics. While the overall surface charge density of tubulin is negative, key regions along the longitudinal dimer-dimer interface (along a single protofilament), and protofilament-protofilament interface (side-to-side between tubulin in two different protofilaments) possess positive regions to promote MT assembly. Zinc is a divalent positive ion, and likely to affect the electrostatic surface. Several key changes were observed in the overall electrostatic profile of tubulin upon addition of zinc (see Figure 6).

The change in region 1 is due to zinc binding at residues βCys241, βCys356, and βAsp357, which is near the colchicine-binding region. The change increases the area's positive potential. In normal protofilament-protofilament interactions this positive region interacts with the negative region of an adjacent dimer. By increasing the positive potential the interaction strength would be increased.

The change in region 2 is due to zinc binding at the MT surface to residues βHis266, βGlu431. Since this region is on the outer surface of the MT, it is not expected to play a role in normal protofilament-protofilament interactions. However, it is expected to strengthen protofilament-protofilament interactions in zinc induce sheet formations due to the anti-parallel protofilament alignment.

The change in region 3 is due to the zinc ion interacting with αHis192, αGlu420, and αAsp424, including one of the key residues involved in zinc-induced tubulin sheet formation. This large change from a negative potential to a positive potential occurs on the outer MT surface, and again is not expected to play a role in normal protofilament-protofilament interactions. However, as with region 2, it would strengthen protofilament-protofilament interactions in the zinc-induced sheet formation.

The change at region 4 is due to the combined effects of zinc at regions 2, 3, and 5. This region normally has a negative potential, which interacts with a positive region on an adjacent protofilament to promote assembly. The increase in positive potential along the interface between α and β tubulin would serve to weaken the binding between protofilaments.

The change at region 5 is due to zinc binding to residues at αAsp69, αGlu71 near the non-exchangeable GTP site on α-tubulin. This region is normally negative, and due to protofilament shift interacts with a negative region on adjacent dimers. By changing the region to a positive potential the protofilament-protofilament interaction would strengthen. However, if both region 1 and region 5 have increased positive potentials due to zinc binding, the protofilament-protofilament interaction would be severely hindered, possibly promoting the transition to the zinc sheet formation.

The change at region 6 is due to the combined effects of the zinc at residues βCys241, βCys356, and βAsp357, as well as the zinc at βHis139. This effect is minor, and not expected to play a significant role in protofilament-protofilament or dimer-dimer interactions.

The change in region 7 is due to the zinc ion near the exchangeable GTP binding region binding with βHis139. This change is minor, and while moving from a negative potential to a more positive potential, it is not expected to induce any significant change in dimer-dimer interactions. However, GTP hydrolysis at this site triggers MT depolymerization, e.g. catastrophes (Figure 1), so the presence of zinc at this site may stabilize microtubule assembly by preventing such events.

**Figure 7:**
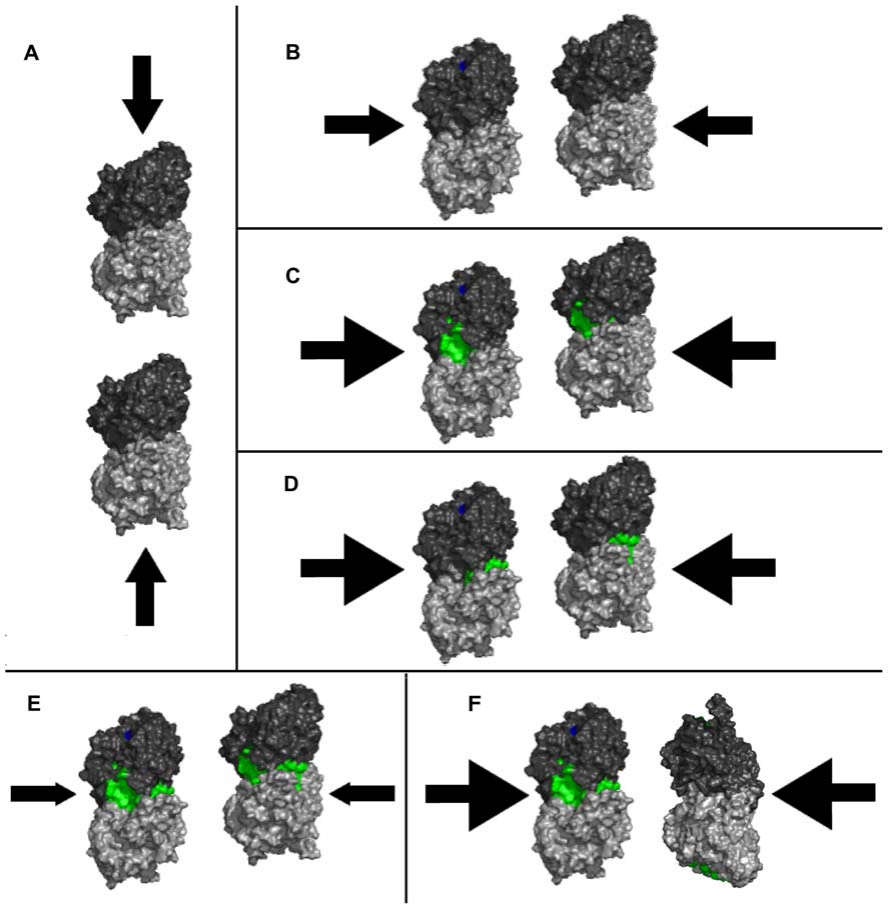
Changes in tubulin interactions due to presence of zinc. In the absence of zinc both longitudinal (dimer-dimer) interactions, and lateral (protofilament-protofilament) interactions, (A) and (B) respectively, behave at baseline (black arrows). Longitudinal interactions are not affected by zinc. Zinc bound to tubulin (affected areas in green) at region 3 or region 5, (C) and (D) respectively, has increased lateral interactions (green arrows). Zinc bound to tubulin at region 3 and region 5, (E), have decreased lateral interactions (red arrows).

Presence of zinc at low to moderate levels is thus expected to increase side-to-side, protofilament-protofilament interactions, stabilizing and promoting MT polymerization, and possibly preventing depolymerization by GTP hydrolysis (Figure 7). However, at higher zinc concentrations protofilament interactions would be hindered, and anti-parallel protofilament orientation would become preferred, promoting transition to zinc-sheet formations.

**Figure 8:**
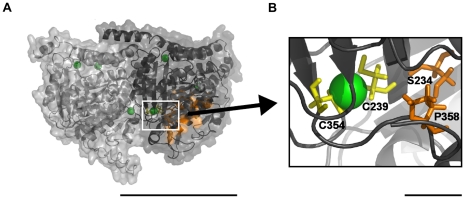
Zinc interaction with the Taxol binding region. (A) Transparent tubulin dimer surface overlaying a cartoon representation. Green Spheres - high stringency predicted zinc-binding sites. Orange – Taxol binding pocket. Scale bar 5 nm. (B) Predicted zinc site bound by cysteine 239 and cysteine 354 (yellow) is within 7 Å of serine 234 and proline 358 of the taxol binding pocket characterized by [37]. Scale bar 5 Å.

As discussed above, AD is known to be associated with abnormal behavior of MAP-tau. With regard to zinc, concentrations of zinc 10- to 30-fold lower than the normal 100–300 µM concentrations found in synaptic vesicles have been found to enhance MAP-tau fibrillization [80]. MAP-tau induces cooperative binding of the chemotherapeutic drug taxol to MTs, and the repeat motifs of MAP-tau stabilize MTs in a way similar to taxol indicating the importance of this region to MAP-tau-MT interactions [81], [82]. Hydroxide ions in solution, or interacting proteins, may react with the predicted zinc-binding site near the taxol-binding region, which is bound by only two cysteine residues (Figure 8). The key interacting regions of the repeat motifs of MAP-tau, which possess a sequence similarity with a region of α-tubulin that normally occupies the taxol-binding site [83], contain a histidine residue capable of interacting with zinc. Additionally, repeat 3 contains an additional histidine within this region. Beyond the sequence similarity region each of the four repeats contains at least one residue capable of binding zinc (glutamic acid in repeat 1, cysteine and aspartic acid in repeat 2, cysteine in repeat 3, and aspartic acid in repeat 4). This suggests that a zinc ion in the taxol-binding region may further stabilize MAP-tau binding to MT. However, the exact structure of and interaction in these binding regions is unknown.

### Kinetic Model of Aβ-induced Zinc Dyshomeostasis Leading to Microtubule Destabilization

Below we develop a simple mathematical model that quantifies the interplay between zinc absorption by Aβ (and a subsequent aggregation [83] with zinc binding by MTs and its utilization in tubulin re-incorporation into MTs. An insufficient supply of zinc would be expected to result in an imbalance in the MT turnover inside neurons leading to their gradual deterioration.

**Figure 9:**
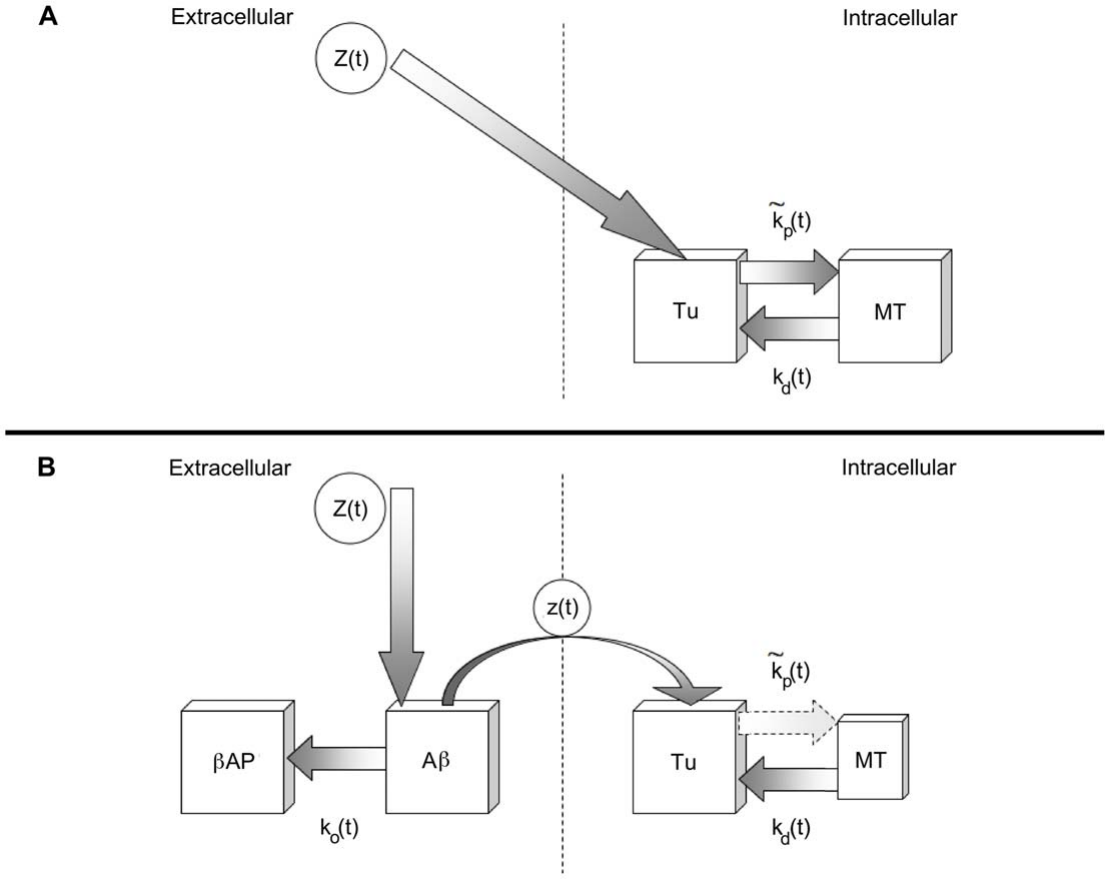
Schematic representation of the multi-compartment model for the kinetic equations governing the dynamics of zinc binding to Aβ and MTs. (A) Normal brain, zinc crosses the membrane and promotes MT polymerization. (B) AD brain, Aβ sequesters zinc promoting plaque formation, while MT polymerization is compromised resulting in a loss of MT.

The model we develop is based on Figure 9 where Aβ resides in the extracellular compartment and satisfies polymerization kinetics catalyzed by zinc. Due to the six orders of magnitudes in the difference between the binding affinities of zinc for Aβ as compared to tubulin, it can be safely assumed that only excess zinc finds its way into the nearby neurons and then into the polymerizing MTs.

First, we assume the total concentration of β-Amyloid protein [A] to be a combination of free Aβ [Aβ] and β-Amyloid Plaque [βAP]:
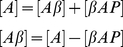
(6.1)Thus,
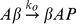
(6.2)where k_o_ is the rate of β-Amyloid oligomerization.

The rate equation describing this reaction is:
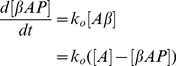
(6.3)The solution of this rate equation is given by:
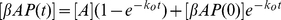
(6.4)where [βAP(t)] is the time dependent concentration of β-Amyloid plaques with [βAP(0)] being the initial concentration of plaques. This will reach a saturation point when the entire concentration of amyloid protein becomes oligomerized into plaques. If zinc has not been completely depleted beyond this saturation point however, the free concentration of zinc z(t), is given by,
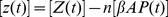
(6.5)where n is the number of zinc binding sites on β-Amyloid plaques, and [Z(t)] is the time dependent zinc influx concentration.

Taking the total concentration of tubulin protein [P] to be a combination of free tubulin protein [Tu] and MTs [MT], i.e.
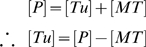
(6.6)we incorporate in the model the fact that zinc increases the polymerization rate of free tubulin into microtubules, k_p_, thus:
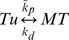
(6.7)where,
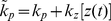
(6.8)and k_z_ is a constant describing how zinc increases tubulin polymerization, [z(t)] is the time dependent zinc concentration after sequestration by Aβ, and k_d_ is the rate at which MTs depolymerize into free tubulin. The rate equation describing this reaction is:
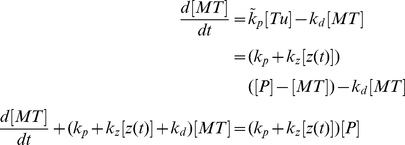
(6.9)The solution of this differential equation can be obtained numerically to give the MT concentration as a function of time [MT(t)].

Taking approximately 10^9^ tubulin dimers polymerized in MTs per neuron, 10^11^ neurons per brain and an average brain volume of 1.3 L gives the average concentration of polymerized tubulin protein in the brain as 130 µM. However, in the neuron this may be ∼3 times higher since glial cells, possessing a MT density much less than neurons, are considered to comprise 50% of the brain. The remaining portion is composed of both neurons and other necessary structures such as ventricles, blood vessels etc. This also does not account for the roughly 20 µM free tubulin dimers in the cytosol. Here, for the purpose of model calculations, we assume the total tubulin protein concentration [P] to be 400 µM.

The MT depolymerization rate constant k_d_ can range between 7 and 89 s^−1^ depending on the buffer conditions [84]. Here, we assume a reasonable value of 70 s^−1^. The polymerization rate constant k_p_ at physiological temperature is given as 9 µM^−1^ s^−1^ and depends on the concentration of free GTP-bound tubulin [84]. Taking the concentration of free GTP-bound tubulin to be 10 µM, slightly above the critical concentration, gives k_p_ = 90 s^−1^.

**Figure 10:**
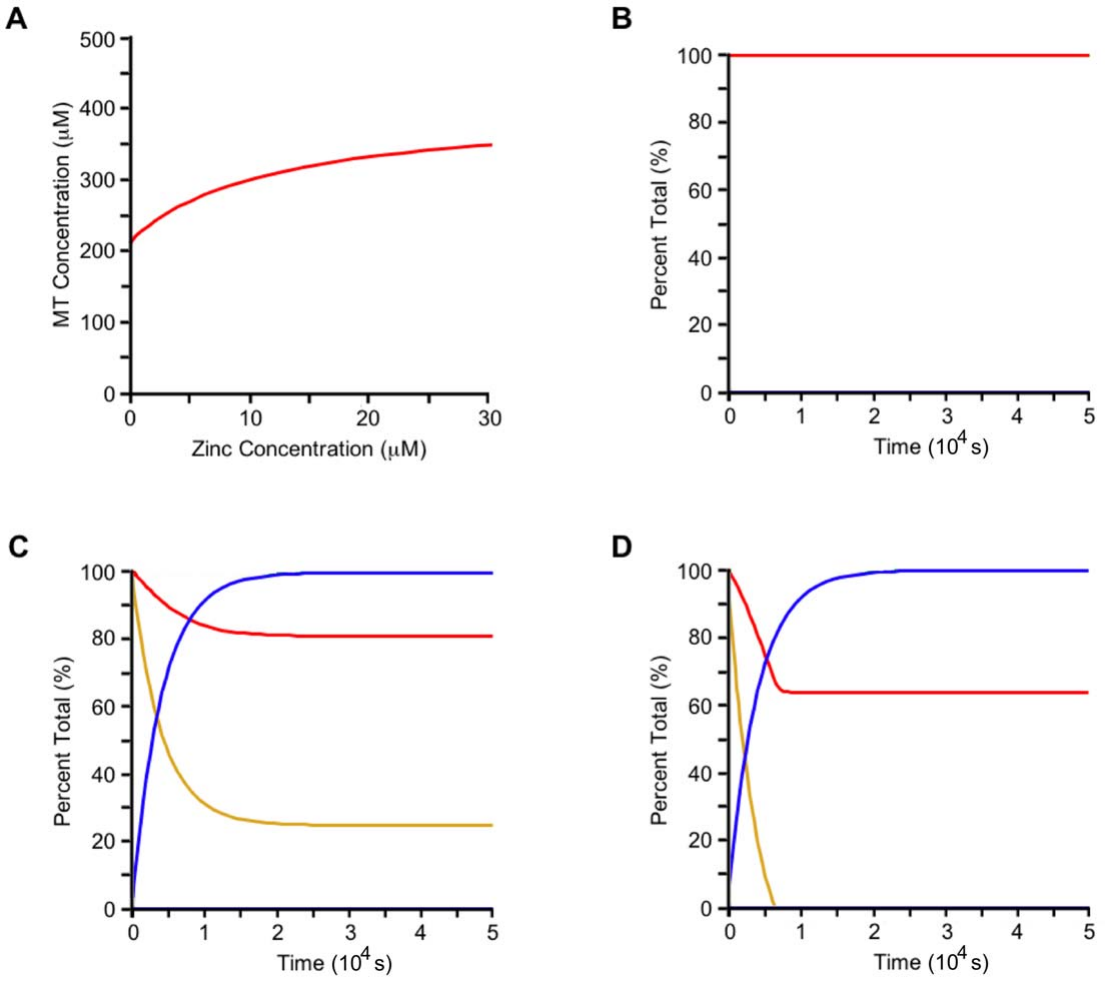
Kinetic model of zinc dyshomeostasis. (A) MT concentration as a function of zinc concentration in the long time limit for k_p_ = 90 s^−1^, k_d_ = 70 s^−1^, and [P] = 400 µM. (B)–(D) Kinetics of β-Amyloid zinc depletion on Microtubule Polymerization. Microtubule (red), β-Amyloid (blue), free zinc after β-Amyloid sequestration (yellow). Parameters: k_p_ = 90 s^−1^, k_d_ = 70 s^−1^, k_z_ = 15 µM^−1^ s^−1^, k_o_ = 2.465(10^−4^) s^−1^, [P] = 400 µM, Z(0) = 20 µM. β-Amyloid protein concentrations for the three cases are, (B) [A] = 0 µM, (C) [A] = 15 µM, and (D) [A] = 25 µM.

With these parameter values and a reasonable zinc-dependent rate constant, k_z_, of 15 µM−^1^ s^−1^, the MT concentration in the absence of zinc is 60% of the value when the zinc concentration is 30 µM (3 times the value of free GTP-bound tubulin). This is comparable to experiment that shows in the absence of zinc the MT concentration is 50% of the concentration when the zinc to tubulin ratio is just below 3∶1, the condition for sheet formation [48]. The long time limit microtubule concentration as a function of zinc concentration, for these parameters, is shown in Figure 10 A.

In the absence of β-amyloid, the polymerized MT concentration remains constant (Figure 10 B). The inclusion of β-amyloid predicts sequestration of the zinc available for tubulin polymerization leading to an overall loss of MTs. In the case where the concentration of β-amyloid is low, free zinc remains available to polymerizing tubulin protein (Figure 10 C). If the concentration of β-amyloid is high, then all zinc is sequestered, and the MT concentration drops to its lowest possible value (Figure 10 D). For the parameters given, and a β-amyloid oligomerization rate constant of 2.465(10^−4^) s^−1^ [85], as little as 25 µM of free β-amyloid per neuron is capable of reducing the overall MT concentration to 64% of its original value, giving a loss of 36% (Figure 10 D). By increasing the depolymerization rate constant, k_d_, to 89 s^−1^, this value can be brought to a 40% loss.

Experimental findings suggest an overall reduction in MT density in AD neurons of 50–55% [2], [86]. Comparable loss can be obtained by increasing the zinc dependent rate constant, k_z_, to 100 µM^−1^ s^−1^, but the exact physiological effect of zinc on tubulin polymerization remains unclear. While these values are comparable the discrepancies do indicate missing factors in the model. This is expected as MT assembly and β-amyloid oligomerization dynamics are kinetically complex, and all factors are not accounted for in this first approximation. However, the general qualitative features of this model indicate that zinc deficiency induced by β-amyloid deposition can result in an overall reduction in MT number independent of PHF formation.

### Metallomic Imaging Mass Spectrometry (MIMS Mapping) of Elemental Zinc Distribution in the Brain of Aged Tg2576 AD Transgenic Mice Compared to Wild Type Controls

**Figure 11:**
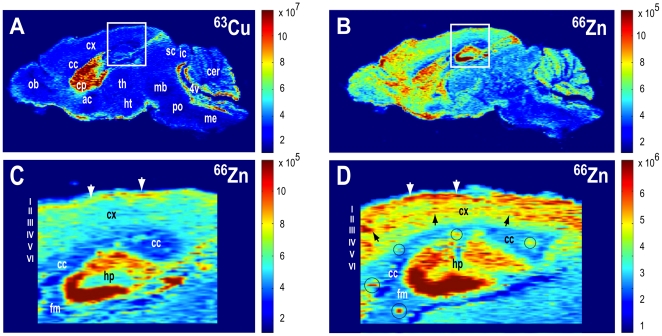
Elemental zinc (^66^Zn) dyshomeostasis in aged Tg2576 AD transgenic mice compared to wild-type littermate controls. (A) Low-resolution metallomic imaging mass spectrometrometry (MIMS) intensity profile of ^66^Zn in a 22-month-old wild-type mouse brain in sagittal section. White box shows detail area of hippocampus, parahippocampal cortex, and major white matter tracts (i.e., corpus callosum, hippocampal fimbria) in panels C and D. An identical zinc distribution profile was observed in the ^70^Zn channel simultaneously obtained during analysis of the same brain section (not shown). (B) Low-resolution metallomic imaging mass spectrometrometry (MIMS) profile of ^63^Cu simultaneously acquired from the same Tg2576 brain section. Note element-specific distribution pattern of ^63^Cu compared to ^66^Zn. (C) High-resolution ^66^Zn MIMS profile of a 22-month-old wild-type mouse hippocampus, adjacent cortex, and parahippocampal fiber tracts. Note prominent laminar distribution of zinc in cortical layer II and hippocampus. (D) High-resolution ^66^Zn MIMS profile of a 22-month-old Tg2576 transgenic mouse brain. Abnormal zinc accumulation is present in all cortical layers, including deep and superficial cortex (black and white arrows, respectively) as well as hippocampus (white hashed ellipse). Pathogenic zinc deposits are also present in white matter (hashed black circles). Regional MIMS profiles of ^66^Zn (66Zn, 65.926 amu, 27.9% abundance) were confirmed by simultaneous profiling of ^70^Zn (69.925 amu, abundance 0.6%; 25 µm resolution) in each brain section. Metallomic imaging mass spectrometry analyses were conducted at the Center for Biometals & Metallomics, Boston University School of Medicine, Boston, MA.

Tg2576 mice over-express human Aβ and age-dependently exhibit Aβ neuropathology and associated neurophysiological deficits that model many aspects of the human clinical disease. Representative ^66^Zn MIMS maps of brains from 22-month-old Tg2576 AD mice and age-matched littermate controls reveal profound AD-specific pathogenic redistribution of zinc in the brains of Tg2576 mice compared to age-matched littermate controls (Figure 11). MIMS mapping revealed AD-linked zinc redistribution and localized pathogenic zinc accumulation within discrete zinc-enriched brain regions (i.e., hippocampus, dentate gyrus, subiculum, and cortical layer II) that are important for memory processing, and especially vulnerable to amyloid-Aβ deposition and AD neuropathology. These data show that zinc dyshomeostasis correlates anatomically with brain areas involved in AD neuropathology, and cognitive dysfunction.

## Discussion

A confirmed diagnosis of AD requires abundant levels of extracellular amyloid plaques composed of Aβ, and intracellular NFTs composed of hyper-phosphorylated tau. While all four of the well-established genes for AD are associated with excessive accumulation of Aβ, NFTs are required for driving cognitive impairment. The mechanism linking excessive β-amyloid to tangle pathology, and the specific mechanism by which NFT result in cognitive impairment remains unknown. Here, we suggest that sequestration of zinc by β-amyloid deposits (Aβ oligomers and plaques) leads to reduced intra-neuronal zinc levels, and in some cases excess zinc, in neighboring neurons. Low zinc, or excessive zinc, then destabilizes microtubules, leading to NFT and cognitive impairment.

To investigate the hypothesis, we:

1. Employed molecular modeling of zinc binding to tubulin, the component protein of microtubules. We find that adequate, but not excessive levels of zinc strengthen side-to-side electrostatic attraction, promoting microtubule lattice stability, and that high levels of zinc promote aberrant interactions.
2. Present a kinetic model of Aβ-induced zinc dyshomeostasis leading to microtubule destabilization.
3. Demonstrate element-specific, anatomically localized zinc dyshomeostasis in specific brain regions of aged Tg2576 AD transgenic mice compared to wild-type littermate controls.

We conclude with an overall hypothesis of AD pathogenesis in which excessive levels of extracellular Aβ in the form of oligomers and amyloid plaques result in low intraneuronal zinc levels. This in turn destabilizes MTs due to reduced side-to-side attractive electrostatic interactions between tubulins (and thus reduced protofilament-to-protofilament interactions). Insufficient zinc may also trigger GTP hydrolysis and MT ‘catastrophes’, which then induce NFT formation and cognitive impairment. Excess zinc also destabilizes MTs thorough aberrant tubulin interactions. This novel hypothesis links β-amyloid pathology to intra-neuronal lesions and cognitive impairment via microtubules.

This hypothesis is consistent with recent experimental evidence. Grabrucker et al. investigate the effects of low/insufficient intracellular zinc due to Aβ sequestration [87]. They found insufficient zinc leads to a reduction in synapse density, and ProSAP2/Shank 3 and Shank 1 protein levels in the postsynaptic density independent from alterations on the transcription level. However, the effect of zinc levels on NFT formation and MT destabilization is not considered. These findings may be the result of hindered MT-based transport of proteins, and a general feature of neurons with insufficient or excess zinc. Kim et al. consider the AD-related effects of excess zinc [88]. They conclude that zinc induces tau Ser214 phosphorylation through the Ras-Raf/mitogen-activated protein kinase kinase/extracellular signal-regulated kinase (ERK) pathway, which interferes with microtubule polymerization. However, as we have discussed, excess intracellular zinc may induce aberrant MT formations leading to an increase in free tau vulnerable to hyperphosphorylation, such as by the ERK pathway. This would indeed explain both the increase in tau phosphorylation, and the MT destabilization.

It has been suggested that the relation between Aβ and NFTs is based in mTOR-dependent signaling [89], with tau phosphorylation regulated by PI3K/mTOR signaling [90]. Aβ accumulation has been shown to increase mTOR signaling [91], suggesting that Aβ may facilitate tau pathology through the mTOR pathway. However, studies at the Karolinska Institutet suggests that both Aβ and increased extracellular zinc can activate mTOR-dependent signaling, resulting in an increase of tau synthesis and phosphorylation [89], [92]–[94]. In fact, the increase in phosphorylated p70S6K levels in differentiated SH-SY5Y cells treated with Aβ42 [95] is approximately consistent with the increase seen in undifferentiated SH-SY5Y cells treated with 100 µM zinc [94]. Further, the mTOR pathway is activated by microtubule damage [96], so mTOR activation may follow Aβ zinc sequestration and microtubule instability.

The hypothesis suggests that effective treatment and prevention of AD could be achieved by approaches aimed at microtubule function and stability. Pharmacologically, this could be a compound that could competitively remove zinc away from Aβ, and redistribute it back to neurons. The 8-hydroxyquinolone and zinc ionophore, PBT2 (Prana Biotechnology) has been reported to carry out this role and ameliorate AD pathology and improve cognition in AD mouse models [97]. Additionally, in a phase 2 clinical trial PBT2 rapidly led to significant cognitive improvement in AD patients [98]. With regard to mechanism of action, PBT2 enters the brain and is then attracted to the extracellular pool of zinc that is in a dissociable equilibrium with Aβ, e.g., in senile plaques and oligomers. PBT2 would then form a ternary complex with zinc and Aβ. The affinity of PBT2 for zinc (10^−12^ to 10^−13^) is several orders of magnitude stronger than that of Aβ. Thus, PBT2 is able to strip zinc away from Aβ leading to reduced aggregation, dissolution of Aβ oligomers (that have not been covalently cross-linked). The drug-zinc complex can then enter the cell leading to redistribution of zinc that was previously trapped by Aβ deposits, e.g. synaptic oligomers and amyloid plaques. PBT2 was able to prevent Aβ oligomer-induced inhibition of LTP and restore dendritic spine density in transgenic mice. While this protective effect could be partially due to the prevention and dissolution of Aβ oligomers, it is also possible that the liberation and redistribution of zinc, which was trapped in Aβ aggregates, was responsible. In support of the latter, Adlard et al. [99] showed that knock out of the zinc transporter, ZnT3, responsible for releasing zinc into the synapse led to impaired LTP and cognitive deficits similar to those observed in AD transgenic mice. Thus, it would be interesting to test whether a zinc ionophore, like PBT2, might also be able to prevent NFT formation in the presence of excess Aβ oligomer and β-amyloid deposits, by redistributing zinc that was previously sequestered by β-amyloid into neurons and onto MT according to the hypothesis proposed in this paper. Redistribution of zinc to MTs may not only prevent tauopathy and NFT formation, but, in a more general context, also ameliorate cognition based on the proposed direct role of MTs in information processing.

In addition to pharmacological intervention, another therapeutic approach to AD suggested by our hypothesis could include energy/field therapies aimed at microtubule polymerization and integrity. Transcranial magnetic, electric and ultrasound therapies are noninvasive techniques being tested for treatment of psychiatric and neurological disorders. Transcranial magnetic stimulation (TMS) has been used for treatment of depression [100], and transcranial electrical stimulation (TES) has been shown to improve memory [101]. Transcranial ultrasound (TUS) is application of mechanical vibrations to the scalp, e.g. in the temporal region, in the range between 20,000 Hz and 30 megahertz, shown to have electrophysiological and behavioral effects in animals [102], [103]. Microtubules have been shown to have resonances precisely in the range of TUS (e.g. 12 kHz to 30 MHz [104]). Thus, TUS may promote microtubule activity, and is a potential therapeutic tool for the treatment of AD.

In summary, we propose a novel hypothesis linking β-amyloid and tangle pathologies in AD. The sequestration of zinc by β-amyloid deposits (Aβ oligomers and plaques) not only drives aggregation of Aβ but leads to local zinc dyshomeostasis and depletion in the vicinity of β-amyloid deposits. This would be predicted to engender reduced and/or excess intra-neuronal zinc levels. Employing molecular modeling of zinc binding to microtubules (MTs) versus Aβ, we demonstrate how insufficient and/or excessive intra-neuronal zinc levels could destabilize microtubules, freeing tau proteins to aggregate in paired helical filament and NFT, leading to neurodegeneration and cognitive impairment. Experimental testing in cell- and animal-based models of AD will be necessary to provide physical evidence supporting this zinc dyshomeostasis hypothesis of AD linking β-amyloid and NFT pathologies.

## References

[1] Tanzi RE, Bertram L (2005). Twenty Years of the Alzheimer's Disease Amyloid Hypothesis: A Genetic Perspective.. Cell.

[2] Cash AD, Aliev G, Siedlak SL, Nunomura A, Fujioka H (2003). Microtubule reduction in Alzheimer's disease and aging is independent of tau filament formation.. Am J Pathol.

[3] Sigurdsson EM (2009). Tau-Focused Immunotherapy for Alzheimer's disease and related tauopathies.. Curr Alzheimer Res.

[4] Bush AI, Tanzi RE (2008). Therapeutics for Alzheimer's disease based on the metal hypothesis.. Neurotherapeut.

[5] Díaz JF, Pantos E, Bordas J, Andreu JM (1994). Solution Structure of GDP-tubulin Double Rings to 3 nm Resolution and Comparison with Microtubules,. J Mol Biol.

[6] Vater W, Böhm KJ, Unger E (1997). Tubulin assembly in the presence of calcium ions and taxol: Microtubule bundling and formation of macrotubule-ring complexes.. Cell Motil Cytoskel.

[7] Unger E, Böhm K, Vater W (1990). Structural Diversity and Dynamics of Microtubules and Polymorphic Tubulin Assemblies.. Electron Microsc Rev.

[8] Dixit R, Ross JL, Goldman YE, Holzbaur ELF (2008). Differential Regulation of Dynein and Kinesin Motor Proteins by Tau.. Science.

[9] Cronly-Dillon J, Carden D, Birks C (1974). The possible involvement of brain microtubules in memory fixation.. J Exp Biol.

[10] Nelson TJ, Backlund PS, Alkon DL (2004). Hippocampal protein-protein interactions in spatial memory.. Hippocampus.

[11] Cavallaro S, D'Agata V, Manickam P, Dufour F, Alkon DL (2002). Memory- specific temporal profiles of gene expression in the hippocampus.. Proc Natl Acad Sci USA.

[12] Bianchi M, Fone KF, Azmi N, Heidbreder CA, Hagan JJ (2006). Isolation rearing induces recognition memory deficits accompanied by cytoskeletal alterations in rat hippocampus.. Eur J Neurosci.

[13] Liu HX, Zhang JJ, Zheng P, Zhang Y (2005). Altered expression of MAP-2, GAP-43, and synaptophysin in the hippocampus of rats with chronic cerebral hypoperfusion correlates with cognitive impairment.. Brain Res Mol Brain Res.

[14] Shimada A, Tsuzuki M, Keino H, Satoh M, Chiba Y (2006). Apical vulnerability to dendritic retraction in prefrontal neurons of ageing SAMP10 mouse: a model of cerebral degeneration.. Neuropathol Appl Neurobiol.

[15] Khuchua Z, Wozniak DF, Bardgett ME, Yue Z, McDonald M (2003). Deletion of the N-terminus of murine MAP2 by gene targeting disrupts hippocampal CA1 neuron architecture and alters contextual memory.. Neurosci.

[16] Woolf NJ (1998). A structural basis for memory storage in mammals.. Prog Neurobiol.

[17] Woolf NJ, Zinnerman MD, Johnson GV (1999). Hippocampal microtubule-associated protein-2 alterations with contextual memory.. Brain Res.

[18] Woolf NJ, Young SL, Johnson GV, Fanselow MS (1994). Pavlovian conditioning alters cortical microtubule-associated protein-2.. Neuroreport.

[19] Woolf NJ (1993). Cholinoceptive cells in rate cerebral cortex: Somatodendritic immunoreactivity for muscarinic receptor and cytoskeletal proteins.. J Chem Neuroanat.

[20] Van der Zee EA, Douma BR, Bohus B, Luiten PG (1994). Passive avoidance training induces enhanced levels of immunoreactivity for muscarinic acetylcholine receptor and coexpressed PKC gamma and MAP-2 in rat cortical neurons.. Cereb Cortex.

[21] Zervas M, Opitz T, Edelmann W, Wanier B, Kucherlapati R (2005). Impaired hippocampal long-term potentiation in microtubule-associated protein 1B-deficient mice.. J Neurosci Res.

[22] Roberts LA, Large CH, Higgins MJ, Stone TW, O'Shaughnessy CT (1998). Increased expression of dendritic mRNA following the induction of long-term potentiation.. Brain Res Mol Brain Res.

[23] Fukunaga K, Muller D, Miyamoto E (1996). CaM kinase II in long-term potentiation.. Neurochem Int.

[24] Hameroff SR, Craddock TJA, Tuszynski JA (2010). “Memory bytes” - molecular match for CaMKII phosphorylation encoding of microtubule lattices.. J Integr Neurosci.

[25] Craddock TJA, Tuszynski JA, Hameroff (2012). Cytoskeletal signaling: Is molecular memory encoded in microtubule lattices by CaMKII phosphorylation?. PLoS Comp Biol.

[26] Hameroff SR, Watt RC (1982). Information processing in microtubules.. J Theor Biol.

[27] Tuszynski JA, Brown JA, Hawrylak P (1998). Dielectric polarization, electrical conduction, information processing and quantum computation in microtubules, are they plausible?. Phil Trans R Soc London [Biol].

[28] Craddock TJA, Beauchemin C, Tuszynski JA (2009). Information processing mechanisms in microtubules at physiological temperature: Model predictions for experimental tests.. Biosystems.

[29] Hagan S, Hameroff SR, Tuszynski JA (2002). Quantum Computation in Brain Microtubules: Decoherence and Biological Feasibility.. Phys Rev E.

[30] Hameroff S, Nip A, Porter M, Tuszynski JA (2002). Conduction pathways in microtubules, biological quantum computation, and consciousness.. Biosystems.

[31] Tuszynski JA, Hameroff SR, Sataric MV, Trpisova B, Nip MLA (1995). Ferroelectric Behavior in Microtubule Dipole Lattices: Implications for Information Processing, Signalling and Assembly/Disassembly.. J Theor Biol.

[32] Woolf NJ, Priel A, Tuszynski JA (2010). Nanotechnology, Nanostructure, and Nervous System Disorders, in Nanoneuroscience: Structural and Functional Roles of the Neuronal Cytoskeleton in Health and Disease.

[33] Thies E, Mandelkow EM (2007). Missorting of tau in neurons causes degeneration of synapses that can be rescued by the kinase MARK2/Par-1.. J Neurosci.

[34] Loring JF, Wen X, Lee JM, Seilhamer J, Somogyi R (2001). A gene expression profile of Alzheimer's disease. DNA Cell Biol. 20: 683–695.. Erratum in: DNA Cell Biol (2002).

[35] Ashford JW, Soultanian NS, Zhang SX, Geddes JW (1998). Neuropil threads are collinear with MAP2 immunostaining in neuronal dendrites of Alzheimer brain.. J Neuropathol Exp Neurol.

[36] Hameroff S (2010). The “conscious pilot”—dendritic synchrony moves through the brain to mediate consciousness.. J Biol Phys.

[37] Nogales E, Wolf SG, Downing KH (1998). Structure of the αβ tubulin dimer by electron crystallography.. Nature.

[38] Löwe J, Li H, Downing KH, Nogales E (2001). Refined Structure of αβ-Tubulin at 3.5 Å Resolution,. J Mol Biol.

[39] Mathie A, Sutton GL, Clarke CE, Veale EL (2006). Zinc and copper: Pharmacological probes and endogenous modulators of neuronal excitability.. Pharmacol Therapeut.

[40] Weiss JH, Sensi SL, Koh JY (2000). Zn2+: a novel ionic mediator of neural injury in brain disease.. Trends Pharmacol Sci.

[41] Koh JY (2001). Zinc and disease of the brain.. Mol Neurobiol.

[42] Frederickson CJ, Koh JY, Bush AI (2005). The neurobiology of zinc in health and disease.. Nat Rev Neurosci.

[43] Frederickson CJ, Smythies JR, Bradley RJ (1989). Neurobiology of zinc and zinc-containing neurons.. International Review of Neurobiology Volume 31.

[44] Adlard PA, Bush AI, Malva JO (2007). Metal Ions and Alzheimer's Disease.. Interaction Between Neurons and Glia in Aging and Disease.

[45] Gaskin F, Kress Y, Brosnan C, Bornstein M (1978). Abnormal tubulin aggregates induced by zinc sulfate in organotypic cultures of nerve tissue.. Neurosci.

[46] Haskins KM, Zombola RR, Boling JM, Lee YC, Himes RH (1980). Tubulin assembly induced by cobalt and zinc.. Biochem Biophys Res Commun.

[47] Hesketh JE (1984). Microtubule assembly in rat brain extracts: Further characterization of the effects of zinc on assembly and cold stability.. Int J Biochem.

[48] Ames BN (2006). Zinc Deficiency and Microtubule Function in Prostate Cells.. DTIC.

[49] Edström A, Mattsson H (1975). Small amounts of zinc stimulate rapid axonal transport in vitro.. Brain Res.

[50] Gaskin F (1981). In vitro microtubule assembly regulation by divalent cations and nucleotides.. Biochem.

[51] Kress Y, Gaskin F, Brosnan CF, Levine S (1981). Effects of zinc on the cytoskeletal proteins in the central nervous system of the rat.. Brain Res.

[52] Hesketh JE (1982). Zinc-stimulated microtubule assembly and evidence for zinc binding to tubulin.. Int J Biochem.

[53] Oteiza PI, Cuellar S, Lonnerdal B, Hurley LS, Keen CL (1990). Influence of maternal dietary zinc intake on in vitro tubulin polymerization in fetal rat brain.. Teratology.

[54] Oteiza PI, Hurley LS, Lonnerdal B, Keen CL (1990). Effects of marginal zinc deficiency on microtubule polymerization in the developing rat brain.. Biol Trace Elem Res.

[55] Wang FD, Zhao FJ, Jing NH (1999). Effect of dietary zinc on microtubule-associated protein 2 expression in the brain of mice.. Acta Physiologica Sinica.

[56] Wang F, Zhao F, Guo J, Jing N (2000). Mechanism of impairment to microtubule polymerization resulting from zinc deficiency during pregnancy and lactation in mice,. J Hygiene Research.

[57] Eagle GR, Zombola RR, Himes RH (1983). Tubulin-zinc interactions: binding and polymerizaton studies.. Biochem.

[58] Hesketh JE (1983). Zinc binding to tubulin.. Int J Biochem.

[59] Melki R, Carlier MF (1993). Thermodynamics of tubulin polymerization into zinc sheets: Assembly is not regulated by GTP hydrolysis.. Biochem.

[60] Banerjee A, Roychowdhury S, Bhattacharyya B (1982). Zinc-induced self-assembly of goat brain tubulin: Some novel aspects.. Biochem Biophys Res Commun.

[61] Prus K, Wallin M (1983). Characterization of acid and alkaline phosphatase activity in preparations of tubulin and microtubule-associated proteins,. FEBS Lett.

[62] Bernstein FC, Koetzle TF, Williams GJ, Meyer EE, Brice MD (1977). The Protein Data Bank: A Computer-based Archival File For Macromolecular Structures.. J Mol Biol.

[63] Sali A, Blundell TL (1993). Comparative protein modelling by satisfaction of spatial restraints.. J Mol Biol.

[64] Phillips JC, Braun R, Wang W, Gumbart J, Tajkhorshid E (2005). Scalable molecular dynamics with NAMD.. J Comp Chem.

[65] DeLano W (2002). PyMOL Release 0.99.

[66] Li H, DeRosier DJ, Nicholson WV, Nogales E, Downing KH (2002). Microtubule Structure at 8 Å Resolution.. Structure.

[67] Sept D, Baker NA, McCammon JA (2003). The physical basis of microtubule structure and stability.. Prot Sci.

[68] Ebert JC, Altman RB (2008). Robust recognition of zinc binding sites in proteins.. Prot Sci.

[69] Ester M, Kriegel HP, Sander J, Xu X, Simoudis E, Han J, Fayyad U (1996). A density-based algorithm for discovering clusters in large spatial databases with noise.. Proceedings of the Second International Conference on Knowledge Discovery and Data Mining.

[70] Li H, Robertson AD, Jensen JH (2005). Very Fast Empirical Prediction and Interpretation of Protein pKa Values.. Proteins.

[71] Dolinsky TJ, Nielsen JE, McCammon JA, Baker NA (2004). PDB2PQR: an automated pipeline for the setup, execution, and analysis of Poisson-Boltzmann electrostatics calculations.. Nucleic Acids Res.

[72] Dolinsky TJ, Czodrowski P, Li H, Nielsen JE, Jensen JH (2007). PDB2PQR: Expanding and upgrading automated preparation of biomolecular structures for molecular simulations.. Nucleic Acids Res.

[73] Baker NA, Sept D, Joseph S, Holst MJ, McCammon JA (2001). Electrostatics of nanosystems: application to microtubules and the ribosome.. Proc Natl Acad Sci U S A.

[74] Pang YP (1999). Novel zinc protein molecular dynamics simulations: steps toward antiangiogenesis for cancer treatment.. J Mol Model.

[75] Pang YP, Xu K, El Yazal J, Prendergast FG (2000). Successful molecular dynamics simulation of the zinc-bound farnesyltransferase using the cationic dummy atom approach.. Protein Sci.

[76] Pang YP (2001). Successful molecular dynamics simulation of two zinc complexes bridged by a hydroxide in phosphotriesterase using the cationic dummy atom method.. Proteins.

[77] Hsiao K, Chapman P, Nilsen S, Eckman C, Harigaya Y (1996). Correlative memory deficits, Abeta elevation, and amyloid plaques in transgenic mice.. Science.

[78] Wandosell F, Serrano L, Hernández MA, Avila J (1986). Phosphorylation of tubulin by a calmodulin-dependent protein kinase.. J Biol Chem.

[79] Shu N, Zhou T, Hovmöller S (2008). Prediction of zinc-binding sites in proteins from sequence.. Bioinformatics.

[80] Mo ZY, Zhu YZ, Zhu HL, Fan JB, Chen J (2009). Low Micromolar Zinc Accelerates the Fibrillization of Human Tau via Bridging of Cys-291 and Cys-322.. J Biol Chem.

[81] Ross JL, Santangelo CD, Makrides V, Fygenson DK (2004). Tau induces cooperative Taxol binding to microtubules.. Proc Natl Acad Sci U S A.

[82] Kar S, Fan J, Smith MJ, Goedert M, Amos LA (2003). Repeat motifs of tau bind to the insides of microtubules in the absence of taxol.. EMBO J.

[83] Bush AI, Pettingell WH, Multhaup G, d Paradis M, Vonsattel JP (1994). Rapid induction of Alzheimer A beta amyloid formation by zinc.. Science.

[84] Sept D, Limbach HJ, Bolterauer H, Tuszynski JA (1999). Chemical Kinetics Model for Microtubule Oscillations.. J Theo Biol.

[85] Sabaté R, Gallardo M, Estelrich J (2005). Temperature dependence of the nucleation constant rate in β amyloid fibrillogenesis.. Int J Biol Macromol.

[86] Paula-Barbosa M, Tavares MA, Cadete-Leite A (1987). A quantitative study of frontal cortex dendritic microtubules in patients with Alzheimer disease.. Brain Res.

[87] Grabrucker AM, Schmeisser MJ, Udvardi PT, Arons M, Schoen M (2011). Amyloid beta protein-induced zinc sequestration leads to synaptic loss via dysregulation of the ProSAP2/Shank3 scaffold.. Mol Neurodegener.

[88] Kim I, Park EJ, Seo J, Ko SJ, Lee J (2011). Zinc stimulates tau S214 phosphorylation by the activation of Raf/mitogen-activated protein kinase-kinase/extracellular signal-regulated kinase pathway.. Neuroreport.

[89] Pei JJ, Hugon J (2008). mTOR-dependent signalling in Alzheimer's disease.. J Cell Mol Med.

[90] Meske V, Albert F, Ohm TG (2008). Coupling of Mammalian Target of Rapamycin with Phosphoinositide 3-Kinase Signaling Pathway Regulates Protein Phosphatase 2A- and Glycogen Synthase Kinase-3β-dependent Phosphorylation of Tau.. J Biol Chem.

[91] Caccamo A, Majumder S, Richardson A, Strong R, Oddo S (2010). Molecular Interplay between Mammalian Target of Rapamycin (mTOR), Amyloid-β, and Tau.. J Biol Chem.

[92] Pei JJ, Björkdahl C, Zhang H, Zhou X, Winblad BJ (2008). p70 S6 kinase and tau in Alzheimer's disease.. Alzheimers Dis.

[93] An WL, Cowburn RF, Li L, Braak H, Alafuzoff I (2003). Up-Regulation of Phosphorylated/Activated p70 S6 Kinase and Its Relationship to Neurofibrillary Pathology in Alzheimer's Disease.. Am J Pathol.

[94] An WL, Bjorkdahl C, Liu R, Cowburn RF, Winblad B (2005). Mechanism of zinc-induced phosphorylation of p70 S6 kinase and glycogen synthase kinase 3beta in SH-SY5Y neuroblastoma cells.. J Neurochem.

[95] Lafay-Chebassier C, Perault-Pochat MC, Page G, Rioux Bilan A, Damjanac M (2006). The immunosuppressant rapamycin exacerbates neurotoxicity of Abeta peptide.. J Neurosci Res.

[96] Calastretti A, Bevilacqua A, Ceriani C, Viganò S, Zancai P (2001). Damaged microtubules can inactivate BCL-2 by means of the mTOR kinase.. Oncogene.

[97] Adlard PA, Cherny RA, Finkelstein DI, Gautier E, Robb E (2008). Rapid Restoration of Cognition in Alzheimer's Transgenic Mice with 8-Hydroxy Quinoline Analogs Is Associated with Decreased Interstitial Aβ.. Neuron.

[98] Faux NG, Ritchie CW, Gunn A, Rembach A, Tsatsanis A (2010). PBT2 Rapidly Improves Cognition in Alzheimer's Disease: Additional Phase II Analyses.. J Alzheimers Dis.

[99] Adlard PA, Parncutt JM, Finkelstein DI, Bush AI (2010). Cognitive Loss in Zinc Transporter-3 Knock-Out Mice: A Phenocopy for the Synaptic and Memory Deficits of Alzheimer's Disease?. J Neurosci.

[100] Allan CL, Herrmann LL, Ebmeier KP (2011). Transcranial magnetic stimulation in the management of mood disorders.. Neuropsychobiology.

[101] Chi RP, Snyder AW (2011). Facilitate Insight by Non-Invasive Brain Stimulation.. PLoS ONE.

[102] Tufail Y, Matyushov A, Baldwin N, Tauchmann ML, Georges J (2010). Transcranial pulsed ultrasound stimulates intact brain circuits.. Neuron.

[103] Yoo SS, Bystritsky A, Lee JH, Zhang Y, Fischer K (2011). Focused ultrasound modulates region-specific brain activity.. Neuroimage.

[104] Sahu S, Hirata K, Fujita D, Ghosh S, Bandyopadhyay A (2012). Radio-frequency-induced ultrafast assembly of microtubules and their length-independent electronic properties.. Nature Mater.

[105] Nunez J, Fischer I (1997). Microtubule-associated proteins (MAPs) in the peripheral nervous system during development and regeneration.. J Mol Neurosci.

[106] Halpain S, Dehlmet L (2006). The MAP1 family of microtubule-associated proteins.. Genome Biol.

[107] Gonzalez-Billaut C, Jimenez-Mateos EM, Caceres A, Diaz-Nido J, Wandosell F (2004). Microtubule-associated protein 1B function during normal development, regeneration, and pathological condition in the nervous system.. J Neurobiol.

[108] Riederer BM (2007). Microtubule-associated protein 1B, growth-associated and phosphorylated scaffold protein.. Brain Res Bull.

[109] Dehmelt L, Halpain S (2005). The MAP2/Tau family of microtubule-associated proteins.. Genome Biol.

[110] Sánchez C, Díaz-Nido J, Avila J (2000). Phosphorylation of microtubule-associated protein 2 (MAP2) and its relevance for the regulation of the neuronal cytoskeleton function.. Prog Neurobiol.

[111] Godert M, Crowther RA, Garner CC (1991). Molecular characterization of microtubule-associated proteins tau and MAP2.. Trends Neurosci.

[112] Lian G, Sheen V (2006). Cerebral development disorders.. Curr Opin Pediatr.

[113] Hoogenraad CC, Akhmanova A, Galjart N, De Zeeuw CI (2004). LIMK1 and CLIP-115: linking cytoskeletal defects to Williams syndrome.. Bioessays.

[114] Hirokawa N, Takemura R (2004). Molecular motors in neuronal development, intracellular transport and diseases.. Curr Opin Neurobiol.

[115] Goldstein LS, Yang Z (2000). Microtubule-based transport systems in neurons: the roles of kinesins and dyneins.. Annu Rev Neurosci.

