# Correction: The Zinc Dyshomeostasis Hypothesis of Alzheimer's Disease

**DOI:** 10.1371/annotation/57c710a6-83ba-444c-a352-b9f60125f2fa

**Published:** 2012-04-03

**Authors:** Travis J. A. Craddock, Jack A. Tuszynski, Deepak Chopra, Noel Casey, Lee E. Goldstein, Stuart R. Hameroff, Rudolph E. Tanzi

There was an error in the Competing Interests statement. The authors would like to apologize for this omission and would like to disclose the following information:

Dr. Tanzi serves as a consultant to, and is shareholder in Prana Biotechnology, LTD, which is developing the Alzheimer's therapeutic compound, PBT2. 

