# Correction: The Zinc Dyshomeostasis Hypothesis of Alzheimer's Disease

**DOI:** 10.1371/annotation/866da844-12ad-4fef-a57e-92a4549e035c

**Published:** 2012-06-15

**Authors:** Travis J. A. Craddock, Jack A. Tuszynski, Deepak Chopra, Noel Casey, Lee E. Goldstein, Stuart R. Hameroff, Rudolph E. Tanzi

There were errors in the legend of Figure 11. The correct figure 11 legend is:

"Figure 11. Elemental zinc dyshomeostasis demonstrated by metallomic imaging mass spectrometrometry (MIMS) in brains from aged Tg2576 AD transgenic mice compared to age-matched non-transgenic littermate control mice. (A) Low-resolution MIMS profile of 63Cu in brain from a 22-month-old non-transgenic control mouse. White box shows detail area in panels C,D. (B) Low-resolution MIMS profile of 66Zn simultaneously acquired from the same brain section shown in panel A. An identical MIMS profile was obtained in the 70Zn channel (not shown). Note element-specific distribution pattern of 66Zn compared to 63Cu. White box shows detail area in panels C,D. (C) High-resolution MIMS profile of 66Zn in brain from a 22-month-old non-transgenic control mouse. 66Zn enrichment is evident in hippocampus and cortex, especially layer II (white arrowheads). (D) High-resolution 66Zn MIMS profile in brain from a 22-month-old Tg2576 transgenic mouse. Pathogenic zinc accumulation is present in hippocampus and throughout the cortex, including deep and superficial layers (black and white arrowheads, respectively). Abnormal zinc deposits are also present in white matter (black circles). Regional MIMS profiles of 66Zn were confirmed by simultaneous 70Zn profiling in each brain section examined. Abbreviations: ac, anterior commissure; cc, corpus callosum; cer, cerebellum; cp, caudatoputamen; cx, cerebral cortex (layers numbered I-VI); fm, hippocampal fimbria; hp, hippocampus; ht, hypothalamus; ic, inferior colliculus; mb, midbrain; me, medulla oblongata; ob, olfactory bulb; po, pons; sc, superior colliculus; th, thalamus, 4V, fourth ventricle. MIMS analyses conducted at the Center for Biometals & Metallomics, Boston University School of Medicine." 

