# Correction: The Zinc Dyshomeostasis Hypothesis of Alzheimer's Disease

**DOI:** 10.1371/annotation/e83fbf22-647f-496f-b27d-5aff26898083

**Published:** 2013-05-06

**Authors:** Travis J. A. Craddock, Jack A. Tuszynski, Deepak Chopra, Noel Casey, Lee E. Goldstein, Stuart R. Hameroff, Rudolph E. Tanzi

There were errors in the Figure 7 legend. The correct legend is:

**Figure 7**: Changes in tubulin interactions due to presence of zinc. In the absence of zinc both longitudinal (dimer-dimer) interactions, and lateral (protofilament-protofilament) interactions, (A) and (B) respectively, behave at baseline (black arrows). Longitudinal interactions are not affected by zinc. Zinc bound to tubulin (affected areas in green) at region 1 or region 5, (C) and (D) respectively, has increased lateral interactions (larger arrows). Zinc bound to tubulin at region 1 and region 5, (E), have decreased lateral interactions (smaller arrows). However, when in the zinc sheet orientation, (F), zinc bound to tubulin at region 1 and region 5 can increase lateral interactions. 

